# Effectiveness of multi-component modular intervention among adults with prehypertension in a village of Dakshina Kannada district - a community-based interventional study – protocol

**DOI:** 10.12688/f1000research.129131.1

**Published:** 2023-06-14

**Authors:** Neneh Feren, Rekha Thapar, B Unnikrishnan, Prasanna Mithra, Nithin Kumar, Ramesh Holla, Darshan BB, Himani Kotian

**Affiliations:** 1 Department of Community Medicine, Kasturba Medical College, Mangalore, Manipal Academy of Higher Education, Manipal, India

**Keywords:** Hypertension, Prehypertension, Randomized controlled trials, Block randomization, Non Communicable diseases, WHO-STEPS.

## Abstract

Introduction:

The Joint National Committee (JNC 7) report on Prevention, Detection, Evaluation, and Treatment of Hypertension, defined "prehypertension," as individuals with a Systolic Blood Pressure (SBP) in the range of 120–139 mmHg and a (diastolic blood pressure) DBP of 80–89 mmHg. Prehypertension is directly linked with hypertension which is a precursor of CVDs. Owing to its high conversion rate to hypertension, it is important to identify individuals with blood pressures in this category and bring about lifestyle modifications in them that can prevent them from being hypertensive and from developing cardiovascular diseases later in life.

Methods:

This randomized controlled trial will be done among the selected pre-hypertensive adults of all genders residing in Kateel Gram panchayat, Dakshina Kannada district, Karnataka. A baseline survey will be done initially to assess the level of prehypertension among the study population. To study the effectiveness of the intervention, 142 individuals will be randomly allocated using block randomization technique to intervention and control groups. A multi-component module (educational intervention) will be developed, validated, and administered to participants in the intervention group, while the control group receives standard care. Each participant will then be followed up once in four months till the end of the study period of one year to assess for changes in SBP, DBP, WHR, BMI, stress levels, and usage of tobacco and alcohol.

Ethics and dissemination:

Institutional Ethics Committee approval was obtained from Kasturba Medical College in Mangalore, India.

The plans for dissemination of findings include presenting at scientific conferences and publishing in scholarly journals.

## Introduction

Hypertension is a modifiable risk factor for cardiovascular diseases (CVD) and a major cause of premature death. Early Identification and prevention lower the risk for the same. ^1^

In 2003, the Joint National Committee in their 7 ^th^ report on the Prevention, Detection, Evaluation, and Treatment of Hypertension introduced a new term ‘Prehypertension’ which included SBP (Systolic blood pressure) ranging from 120 – 139 mmHg and DBP (Diastolic blood pressure) from 80 – 89 mmHg. This was a redefined new criterion to increase the emphasis on the excess risk factors associated with BP in this range and to bring public attention to the importance of the prevention of hypertension among all genders. ^2^^–^^4^

Globally, the prevalence of prehypertension is 31% with a high conversion rate to hypertension of 30%. ^2^ It is responsible for almost 62% of cardiovascular and 49% of ischemic heart diseases. ^5^ The prevalence of pre-hypertension in the Indian subcontinent from a study conducted in 2016 was approximately 47% among young urban residents. ^6^ A prevalence of 31.5% was observed in South India, 30% in West India, 24.6% in North India, and 20.9% in East India ^7^

Prehypertension is directly linked with hypertension which is a precursor of CVDs. Owing to its high conversion rate to hypertension, it is important to identify individuals with blood pressures in this category and bring about lifestyle modifications in them that can prevent them from being hypertensive and from developing cardiovascular diseases later in life.

Studies demonstrate that multiple lifestyle modifications lower blood pressure by modifying the risk factors which control hypertension. ^8^ The nutritional requirements of a pre-hypertensive individual can be addressed by adopting a DASH (Dietary Approaches to Stop Hypertension) diet. This diet promotes the reduced intake of salt, incorporating fruits, vegetables, micro-nutrients, and lean meat instead of full-fat meat, low-fat dairy, nuts, and legumes. ^9^

Physical Inactivity and a sedentary lifestyle are major risk factors for hypertension. ^10^ According to multiple studies, exercise training lowers blood pressure in about 75% of the individuals diagnosed with hypertension, with systolic blood pressure and diastolic blood pressure reductions of 11 and eight mmHg, respectively. ^10^ Another risk factor for hypertension is salt consumption. Salt being the main source of sodium in our diet, is usually consumed in large quantities and often ends up being two times higher than the recommended quantity. Reducing salt intake has been identified as one of the most cost-effective measure to reduce the risk of cardiovascular diseases, stroke, and coronary artery disease. ^11^

Tobacco use is one of the biggest public health threat world has ever faced and is one of the major causes of cardiovascular diseases. ^12^ There is a causal relationship between the harmful use of alcohol and increased levels of blood pressure which poses it as a risk factor. ^13^ Several studies have also observed the causal association between high levels of stress and high blood pressure measurements. ^14^

The early identification of individuals with prehypertension along with risk behaviors is important to reduce the burden of non-communicable diseases. Effective non-pharmacological interventions, health education, and lifestyle modifications have been used extensively for lowering the blood pressure among the pre-hypertensive individuals

### Research gaps identified

Even though a high prevalence of pre-hypertension is observed in India, there is a paucity of literature on its prevalence in rural areas of South India. Also, adequate emphasis is not given to the importance of halting progression of pre-hypertension blood pressure levels to hypertension levels in preventive programs. The present study is undertaken to fill the gap in existing information on the effectiveness of multi-component intervention in preventing the progression of pre-hypertension to hypertension among adults in a rural area of South India.

### Review of literature

The term “pre-hypertension” was used by the Joint National Committee's Seventh Report to describe a group of people who have elevated blood pressure and a greater burden of other risk factors, like obesity, diabetes, dyslipidemia, and coronary artery disease. ^2^^–^^4^

In the Cross-sectional study conducted by McNiece KL et al, the prevalence of prehypertension and hypertension was 15.7% and 3.2% respectively. The factors like risk for overweight or overweight along with the racial trait and gender were associated with pre-hypertension independently. ^14^

In a cross-sectional study done by Parek A *et al.* in Vadodara, the prevalence of prehypertension was found to be 24.2%. The mean systolic and diastolic blood pressures were directly proportional to age. The study concludes that periodic screening of people, especially high-risk people regularly, can help in the early detection of hypertension and thus prevent cardiovascular diseases. ^15^

In the study conducted by Parthaje PM on the prevalence of prehypertension in adults in urban South India, blood pressure levels in the prehypertensive range were found in 343 (55%) of the total 643 adults and 185 (29.6%) had previously undiagnosed hypertension. Among the study subjects, a higher proportion were females (69.1) and people in the age group 20 to 39 years (40.5%). A high prevalence of prehypertension was found among the study population ^3^

A study, done by Pimenta E. *et al* which evaluated the effects of pre-hypertension concluded that all prehypertensive patients should get non-pharmacological treatment with lifestyle changes such as weight loss, dietary changes, and an increase in daily physical activity because these measures significantly lower the risk of cardiovascular events. ^16^

In an interventional trial conducted by Darviri C. *et al.* to examine the effectiveness of an 8-week health promotion program consisting of multiple lifestyle modifications intended to lower blood pressure (BP) in pre-hypertensive and patients with hypertension, a significant decline in the blood pressures and anthropometry measurements in the intervention arm was observed when compared to the control arm. ^17^

In a randomized controlled trial studying the importance of multiple lifestyle modifications for patients with prehypertension and hypertension, the intervention tool included health education modules regarding various food pattern changes and increased physical activity. The lifestyle modification and follow-up lasted for two months. The study concluded that combination of various lifestyle interventions, physical activity, and dietary interventions diminished blood pressure and reduced CVD events. ^18^

A study conductedby Rubinstein A in Latin American countries, showed that after 12 months of mHealth (mobile Health) interventions consisting of diet and physical activity among prehypertensives, there was an increase in daily intake of fruits and vegetables and a decrease in daily dietary fat, refined sugar, and high sodium post intervention. A reduction in body weight was also observed at the end of the study period. ^19^

### Aim

To study the effectiveness of the multi-component modular intervention on pre-hypertension among adults in a rural area of Dakshina Kannada District in Karnataka.

### Objectives

The objectives are:

- To develop a comprehensive multi-component module for people with pre-hypertension.
- To assess the effectiveness of modular interventions among prehypertensive individuals.

## Methods

### Background information on the study area

The study will be conducted in the three selected villages of Kateel, a temple town in the Moodubidri taluk ^20^ of Dakshina Kannada District of Karnataka. The population of all the selected villages within Kateel Gram panchayat, according to the census India 2011 is 4470 with 2378 in Kondemula (literacy rate of 87.87%), 1454 in Nadugodu (literacy rate: 85.82%) and 658 in Kilinjur (89.84%). ^21^

### Study design

The initial baseline survey estimated 32% prevalence of prehypertension. One forty-two hypertensive individuals, as per the sample size calculation will be included in the trial.

This will be an open-label study RCT with the parallel group. The trial will be reported along with the Consolidated Standards of Reporting Trials (CONSORT). ^22^

The protocol for this study is reported along with Standard Protocol Items: Recommendations for Interventional Trials (SPIRIT) Guidelines. The Reporting guideline criteria include a completed SPIRIT checklist. ^23^

### Study participants

The study population will be selected from the prehypertensive individuals identified during the baseline by simple random sampling.

### Duration of the study

The study will be conducted for one year from June 2021 to Dec 2022.

### Sample size calculation

A sample size of 142 prehypertensive individuals (71 in each group with 1:1 allocation) was calculated using the formula mentioned below and considering a difference in change score of 0.37 (standard deviation- 0.88) ^19^ in SBP between the intervention and control groups, assuming a clinically acceptable difference of 0.5 irrespective of gender, 95 % confidence interval, 90% power, and along with 10 % non-response error

$$N=2\times {\left(Z_{1-\alpha /2}+Z_{1-\beta }\right)}^{2}\;X\;S^{2}/{\left(\delta –\delta_{0}\right)}^{2}$$

Where Z 1-α =1.96 for a 95% confidence interval, Z1-β = 1.281 for 90% power. δ - δ0 = 0.5 (clinically acceptable difference). S is the combined standard deviation.

### Eligibility criteria

Adults of all genders above the age of 18 who are diagnosed with prehypertension will be eligible to participate. Pregnant women, individuals diagnosed with hypertension, genetic disorders/disabilities, and those who are bedridden will be excluded. Those who do not give consent will also be excluded from the study.

### Sampling method and randomization technique

Simple random sampling will be used to select the study participants from the prehypertensive individuals identified at the baseline assessment. In this randomized controlled Trial (RCT), 142 individuals irrespective of gender, who are pre-hypertensives will undergo random allocation to the intervention and control group. i.e. intervention (A) and control group (B). Applying the block randomization technique, for allocating the 142 participants into two groups 36 blocks are needed, with 4 participants in each block (36 blocks x 4 participants =144 participants).

For getting a block of four participants with an equal no of participants from the intervention & control group the six possible permutations are AABB, BBAA, ABAB, BABA, ABBA & BAAB, which will be numbered as one, two, three, four, five and six respectively. To begin with, a random permutation numbered from one-six will be selected using the lottery technique and the first four participants will be allocated according to the selected permutation. The participants will be allocated into either group in a batch of four.

### Intervention procedure

A health education module will be developed based on the inputs from the subject experts, an extensive literature review, and in-depth interviews with the local stakeholders. This will be a multi-component audio-visual module with relevant textual information.

The creation and curation of the educational module will be done in the vernacular language and local context. Before the modules are used in the study environment, their content validity will be assessed. It will contain four parts.

**Part I** will include general knowledge about hypertension, its causes, and risk factors. It will emphasize the importance of treating it on a prehypertensive level and preventing the conversion to hypertension.

**Part II** will include pictorial demonstrations of various dietary modifications, the food that they should avoid to prevent the progression of prehypertension to hypertension, and the food that they should eat more to improve their health.

**Part III** will be about the importance of physical activities, their benefits, different types of exercises, and the guidelines to follow them.

**Part IV** will consist of different kinds of stress and anxiety relieving techniques and the guidelines to follow them.

Three sessions of modular intervention with a duration of 20 minutes each will be administered to the pre-hypertensive individuals in the intervention group at an interval of three months. Sessions will be conducted in groups at the Rural Health Training Centre of Kasturba Medical College at Kateel. The participants missing one intervention session will be contacted and will be rescheduled with a different group. Participants in the study who miss two or more sessions will be deemed to have dropped out.

### Instruments used for data collection

A Pretested, content validated and semi-structured questionnaire will be used. The instrument will include the following sections: A) General participant information B) Knowledge about high blood pressure, C) WHO STEPS Questionnaire ^24^, D) Perceived Stress Scale ^25^, E) Socio-economic scale ^26^

### Data collection methodology

The study area will be visited on a pre-informed date. All the selected individuals (n=142) with prehypertension from the three villages of Kateel will be included. Participants will be then approached in their houses. They will be explained in their vernacular language regarding the objectives of the study and a participant information sheet will be provided to each one of them which consists of details regarding any queries on dropping out. Written informed consent will be obtained from the participants who are willing to take part. Each eligible participant will be interviewed, and their anthropometric measurements and blood pressure will be measured using standard methods. ^24^ Stress levels will be assessed. It will be in the form of a 5-point Likert scale will be used. The responses ranging from strongly agree (5) to strongly disagree (1) will be recorded. The control group will receive standard patient care.

The intervention and control groups will be followed up every four months. On each visit, the blood pressure and anthropometry of the intervention group will be recorded at Rural Health Training Centre (RHTC), Kateel. The study participants allocated to the control group will be followed up in their houses.

A weekly track record of diet, physical activity, and stress reduction activities will be maintained by the participants. This will be verified by the investigator during each intervention session. At the end of the fourth follow-up session, an assessment will be done concerning their perception of hypertension, perceived stress, usage of tobacco and alcohol, and anthropometric measurements.

CONSORT diagram ^22^


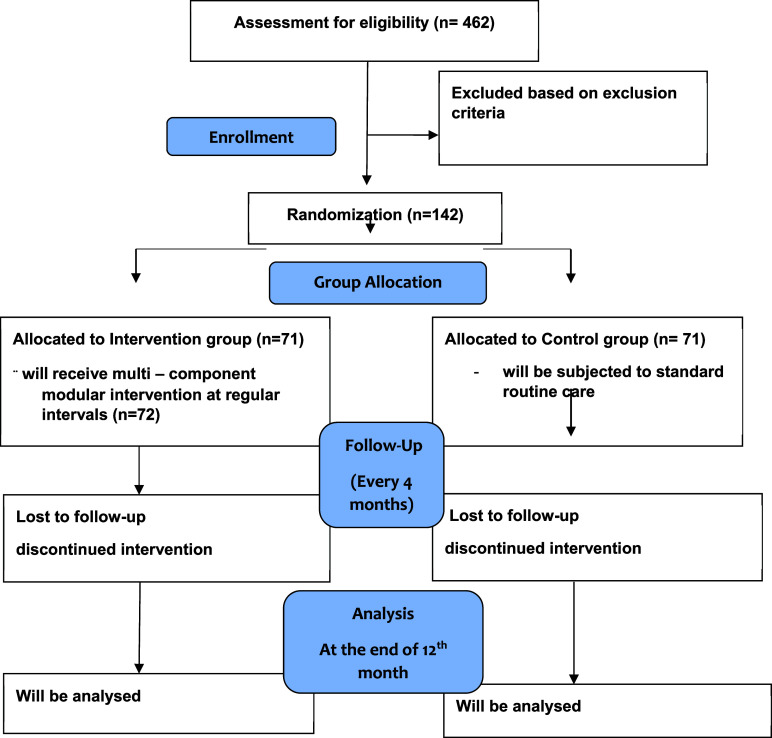


### Test procedures

Measurement of weight ^24^

A portable digital weighing scale will be used to measure weight. The person will be requested to take off their shoes, slippers, sandals, and socks before being instructed to step one foot onto either side of the scale. The instructions will be given to the participant to stand still with their arms at their sides and to face forward. On the instrument, the weight will be measured in kilos to the nearest 0.1 cm. After each use, the scale will be reset to zero.

Measurement of height ^24^

A portable length measuring board will be used to measure height. The participant will be required to take off their shoes, slippers, sandals, and/or headgear which includes a hat, cap, hair bows, comb, etc It will be measured over a thin fabric. The participant will be instructed to face the investigator while standing on the board. The contestant must stand with their feet together, their heels pressed up against the backboard, and their knees straight. The subject is instructed to maintain a straight-ahead gaze with eyes level with ears. By lowering the measuring arm to the patient's head, the reading will be measured to the nearest 0.1 cm. ^24^

Body mass index ^24^

The formula to calculate BMI will be

$$\mathrm{BMI}=\text{Weight}\;\left(\text{in}\;\mathrm{kg}\right)/\left\{\text{height}\;{\left(\text{in meters}\right)}^{2}\right\}$$

Asian BMI Classification:

< 18.5 kg/m ^2^ = underweight

>18.5-22.9 kg/m ^2^ = ideal

>23-24.9 kg/m ^2^ = overweight

> 25 kg/m ^2^ = obese

Measurement of waist-circumference ^24^

A tape with consistent tension will be used to measure waist circumference. It will be measured at the midpoint between the bottom of the last rib and the top of the iliac crest at the end of regular respiration while the arms are relaxed at the sides. The participant will be instructed to wrap the tape over themselves, and the investigator will assist them in positioning it properly. The subject will be instructed to stand with their feet together, their weight spread evenly over both feet, and

their arms relaxed by their sides. Only one reading of the measurement will be made at the level of the tape, to the closest 0.1 cm.

Measurement of hip-circumference ^24^

Hip circumference will be measured using a constant tension tape. The measurement will be taken with the subject minimally clothed. The individual is instructed to stand with their feet together, their weight evenly spread over both feet, and their arms at their sides. The tape would be positioned horizontally over the maximum circumference of the buttocks. Measurement will be read at the level of the tape to the nearest 0.1 cm. It will be measured only once. ^24^

Waist - hip ratio (WHR) ^24^

WHR will be calculated using the formula

WaistHipRatio=waist circumference/hipcircumference.

As recommended by the WHO,

$$<0.9-\text{Normal}>0.9-\text{High}\;\left(\mathrm{Men}\right)$$

$$<0.85-\text{Normal}>0.85-\text{high}\;\left(\text{Women}\right)$$

Blood pressure measurement ^24^

Using a digital automatic blood pressure monitor, blood pressure will be measured. Participants will be requested to sit on a chair with their feet flat on the floor, their legs uncrossed, and their backs supported. At the level of the heart, the cuff will be placed on the patient's uncovered upper arm. It will be placed over the brachial artery. A maximum of two fingers should fit between the distal portion of the cuff and the skin when it is properly positioned. A few centimeters (one-two) above the antecubital fossa will be where the distal portion of the cuff is placed. After detecting a pulse, the monitor will begin taking measurements.

### Outcome measures

• Mean change in blood pressure measurements (SBP, DBP)
• Change in mean BMI, waist circumference and hip circumference, WHR
• Difference in the usage of tobacco and alcohol
• Change in mean stress levels.

### Data management

The collected data will be entered into an excel sheet. After the entry, the data will be cleaned and the missing information will be obtained by reaching out to the study participant. Validation of the proforma will be checked with 10% of the data obtained. A committee for data management will not be formed. The data will be kept confidential with password protection. It will be coded and analyzed using the software ‘IBM Corp. Released 2017. IBM SPSSStatistics for Windows, Version 25.0. Armonk, NY: IBM Corp. ^27^

### Data analysis

Results will be expressed as proportions and summary measures (Mean±SD). Appropriate tables and figures will be used. Intention to treat analysis (ITT) will be followed.

The variables will be compared across the prehypertensive and the normotensive groups using the chi-square test. The factors associated with prehypertension will be studied using Binary logistic regression and multivariate logistic regression. The ‘Hosmer and Lemeshow goodness-of-fit test will be used to assess the fit of the logistic model. A P value < 0.05 will be considered a significant association between predictive and outcome variables (Prehypertension). The confidence intervals (95%) for both the unadjusted and adjusted odds ratios will be reported.

The baseline and post-intervention values across the intervention and control groups will compare using the Mann- Whitney U test for the data that are non-normally distributed and an independent t-test will be used for the normally distributed data (SBP, DBP, BMI, WHR, and stress levels) and chi-square test (Usage of Alcohol and Tobacco). The change score within the group will be compared using repeated measures of ANOVA (WHR) and Friedman’s test (SBP, DBP, BMI, and Stress). A 'p-value less than < 0.05 will signify statistical significance.

### Implications

‐ The findings of this study may help in prioritizing the resources towards the reduction of NCD risk factors.
‐ The multi-component module developed can be used for conducting health education sessions among pre-hypertensive adults in rural areas of South India.

### Study status

Currently, the participants have been recruited and two sessions of multi-modular comprehensive intervention have been conducted for the participants in the intervention group.

### Ethical consideration

IEC (Institutional Ethics Committee) of Kasturba Medical College, Mangalore has approved the study (IECKMCMLR-12/2020/399)

Any changes in the protocol during the course of the study will be submitted to the IEC and approval will be obtained.

This trial is registered prospectively in CTRI India (CTRI/2021/11/037852). Necessary permissions will be obtained from the district health authorities.

A participant information sheet will be administered to all participants. Participants who agree to take part in the study will be asked for a written statement of informed consent. The participant information data will be kept in confidence.

Data Monitoring

Interim analysis will be conducted at 6 months after the initiation of the study. Trial conduct audit will be carried out by the investigators at regular interval of 3 months. No known adverse effect is associated with this study. A separate Data Monitoring Committee (DMC) will not be constituted. The modular intervention will be administered among the participants in the control group at the end of the study.

## Author Contributions

**Neneh Feren**

Roles: Conceptualization, Validation, Writing – Original Draft Preparation, Writing – Review & Editing

**Rekha Thapar**

Roles: Conceptualization, Supervision, Writing – Original Draft Preparation, Writing – Review & Editing

**Prasanna Mithra**

Roles: Methodology, Supervision, Writing – Original Draft Preparation, Writing – Review & Editing

**Nithin Kumar**

Roles: Methodology, Supervision, Writing – Original Draft Preparation, Writing – Review & Editing

**Ramesh Holla**

Roles: Methodology, Supervision, Writing – Original Draft Preparation, Writing – Review & Editing

**Darshan BB**

Roles: Methodology, Supervision, Writing – Original Draft Preparation, Writing – Review & Editing

**Himani Kotian**

Conceptualization, Data Curation, Software, Review and Editing

## Data Availability

No underlying data is associated with this article Open Source Framework: Effectiveness of multi-component modular intervention among adults with prehypertension in a village of Dakshina Kannada district - a community-based interventional study – protocol https://osf.io/ywsj4/?view_only=2fb4312ecb09483d872ce2e0ebbd14dc The supplementary materials available are:
- Informed consent form and participant information sheet - Questionnaire - SPIRIT checklist - Consort diagram SPIRIT checklist. Open Science Framework. EFFECTIVENESS OF MULTI-COMPONENT MODULAR INTERVENTION AMONG ADULTS WITH PREHYPERTENSION IN A VILLAGE OF DAKSHINA KANNADA DISTRICT - A COMMUNITY-BASED INTERVENTIONAL STUDY – PROTOCOL. DOI: https://doi.org/10.17605/OSF.IO/YWSJ4 Data are available under the terms of the Creative Commons Attribution 4.0 International license (CC-BY 4.0).
