# Supplementary material for: Effectiveness of multi-component modular intervention among adults with prehypertension in a village of Dakshina Kannada district - a community-based interventional study – protocol
Source: F1000Res. 2023 Sep 22;12:667. Originally published 2023 Jun 14. [Version 2] doi: 10.12688/f1000research.129131.2 (PMC10993661; doi:10.12688/f1000research.129131.2)
Supplement: Peer review history

# Reviewer report: Reviewer response for version 2

**DOI:** 10.5256/f1000research.155720.r208639

**Published:** 2023-10-03

**Authors:** Hon Lon Tam

**Affiliations:** 1 The Nethersole School of Nursing, The Chinese University of Hong Kong, Hong Kong, Hong Kong

No new concern is raised in the revised version.

Is the study design appropriate for the research question?

Yes

Is the rationale for, and objectives of, the study clearly described?

Partly

Are sufficient details of the methods provided to allow replication by others?

Partly

Are the datasets clearly presented in a useable and accessible format?

Not applicable

Reviewer Expertise:

Cardiometabolic diseases, controlled trial, systematic review, meta-analysis, gerontological nursing

I confirm that I have read this submission and believe that I have an appropriate level of expertise to confirm that it is of an acceptable scientific standard.

# Reviewer report: Reviewer response for version 2

**DOI:** 10.5256/f1000research.155720.r208638

**Published:** 2023-09-25

**Authors:** Karani Magutah

**Affiliations:** 1 Moi University, Eldoret, Uasin Gishu County, Kenya

I have no further comments

Is the study design appropriate for the research question?

Partly

Is the rationale for, and objectives of, the study clearly described?

Yes

Are sufficient details of the methods provided to allow replication by others?

Partly

Are the datasets clearly presented in a useable and accessible format?

Yes

Reviewer Expertise:

Medical physiology and epidemiology

I confirm that I have read this submission and believe that I have an appropriate level of expertise to confirm that it is of an acceptable scientific standard.

# Reviewer report: Reviewer response for version 1

**DOI:** 10.5256/f1000research.141793.r178670

**Published:** 2023-07-06

**Authors:** Hon Lon Tam

**Affiliations:** 1 The Nethersole School of Nursing, The Chinese University of Hong Kong, Hong Kong, Hong Kong

Abstract:

 The use of abbreviation in abstract is not a common practice. CVD was not introduced before the first use. Please add a section to describe the significance and contribution of the study to current research and practice. 

 Introduction

1. The use JCN7 was good, but the it was very old. Also, none of the cited Reference #2-4 was the direct JNC7 document. There are many updated hypertension guidelines you can refer to (1-3).
2. You mentioned lifestyle modifications were important to pre-hypertension people. Please summarize the points as it was too long now.
3. Please summarize the prevalence of pre-hypertension from various studies.
4. You may mention that many people with hypertension did not adhere to healthy lifestyles (4). You can use this point to link with the development of interventions for pre-hypertensive people.
5. You described that several studies had shown the effectiveness of using multi-components to improve blood pressure and lifestyles among pre-hypertensive people, What was the value/significance of current study?

Methods:

1. Where are the villages, South, North, East or West India? Any specific reasons to select these villages?
2. The study had been done in Dec 2022. Why to publish a study protocol, but not the study findings?
3. "For getting a block of four participants with an equal .... The participants will be allocated into either group in a batch of four." Might not be necessary.
4. Please describe the development of the intervention. Any guideline to follow the development of the content? 
5. What will be the expected group size? Who delivered the education? What was the interval between each session, daily, weekly?
6. It was unclear that there were 4 modules, but only 3 sessions. Please describe with modules will be delivered in session 1, 2, and 3.
7. How to control subject contamination between intervention and control groups?
8. Please update blood pressure measurement. It has been suggested to measure at least twice in one measurement (1-3). 
9. Who collect the data from the participants? Who assign the participants to groups?
10. The use of ITT is good, but how to treat missing data?
11. The change score within the group will be compared using repeated measures of ANOVA and Friedman’s test. Any test to examine the change between groups (intervention vs control)?

Implications:

 Please use paragraph format. Urban-rural disparities in hypertension management has been shown globally for years (4, 5, 6). The authors need to make further discussion.

 Figure:

 There was only 71 participants assigned to intervention group, but 72 will receive the modules?

 Suggested references:

1. Unger T, Borghi C, Charchar F, et al. 2020 International Society of Hypertension Global Hypertension Practice Guidelines. Hypertension. 2020;75(6):1334-1357.
2. Whelton PK, Carey RM, Aronow WS, et al. 2017 ACC/AHA/AAPA/ABC/ACPM/AGS/APhA/ASH/ASPC/NMA/PCNA Guideline for the Prevention, Detection, Evaluation, and Management of High Blood Pressure in Adults: A Report of the American College of Cardiology/American Heart Association Task Force on Clinical Practice Guidelines. Journal of the American College of Cardiology. 2018;71(19):e127-e248.
3. Williams B, Mancia G, Spiering W, et al. 2018 ESC/ESH Guidelines for the management of arterial hypertension. European Heart Journal. 2018;39(33):3021-3104.
4. Tam HL, Chair SY, Leung ISH, Leung LYL, Chan ASW. US Adults Practicing Healthy Lifestyles Before and During COVID-19: Comparative Analysis of National Surveys. JMIR Public Health and Surveilance. Mar 31 2023;9:e45697.
5. Ba HO, Camara Y, Menta I, et al. Hypertension and Associated Factors in Rural and Urban Areas Mali: Data from the STEP 2013 Survey. International Journal of Hypertension. 2018:6959165.
6. Ratovoson R, Rasetarinera OR, Andrianantenaina I, Rogier C, Piola P, Pacaud P. Hypertension, a Neglected Disease in Rural and Urban Areas in Moramanga, Madagascar. PLoS One. 2015;10(9):e0137408.

Is the study design appropriate for the research question?

Yes

Is the rationale for, and objectives of, the study clearly described?

Partly

Are sufficient details of the methods provided to allow replication by others?

Partly

Are the datasets clearly presented in a useable and accessible format?

Not applicable

Reviewer Expertise:

Cardiometabolic diseases, controlled trial, systematic review, meta-analysis, gerontological nursing

I confirm that I have read this submission and believe that I have an appropriate level of expertise to confirm that it is of an acceptable scientific standard, however I have significant reservations, as outlined above.

## References

[1] : 2020 International Society of Hypertension Global Hypertension Practice Guidelines. *Hypertension* .2020;75(6) : 10.1161/HYPERTENSIONAHA.120.15026 1334-1357 10.1161/HYPERTENSIONAHA.120.15026 32370572

[2] : 2017 ACC/AHA/AAPA/ABC/ACPM/AGS/APhA/ASH/ASPC/NMA/PCNA Guideline for the Prevention, Detection, Evaluation, and Management of High Blood Pressure in Adults: A Report of the American College of Cardiology/American Heart Association Task Force on Clinical Practice Guidelines. *Hypertension* .2018;71(6) : 10.1161/HYP.0000000000000065 e13-e115 10.1161/HYP.0000000000000065 29133356

[3] : 2018 ESC/ESH Guidelines for the management of arterial hypertension. *Eur Heart J* .2018;39(33) : 10.1093/eurheartj/ehy339 3021-3104 10.1093/eurheartj/ehy339 30165516

[4] : US Adults Practicing Healthy Lifestyles Before and During COVID-19: Comparative Analysis of National Surveys. *JMIR Public Health Surveill* .2023;9: 10.2196/45697 e45697 10.2196/45697 36940169 PMC10131672

[5] : Hypertension and Associated Factors in Rural and Urban Areas Mali: Data from the STEP 2013 Survey. *Int J Hypertens* .2018;2018: 10.1155/2018/6959165 6959165 10.1155/2018/6959165 29610681 PMC5828104

[6] : Hypertension, a Neglected Disease in Rural and Urban Areas in Moramanga, Madagascar. *PLoS One* .2015;10(9) : 10.1371/journal.pone.0137408 e0137408 10.1371/journal.pone.0137408 26355997 PMC4565657

# Response

**Published:** 2023-09-06

**Authors:** Rekha Thapar

**Affiliations:** Kasturba Medical College Hospital, India

1.The use of abbreviation in abstract is not a common practice. CVD was not introduced before the first use. Please add a section to describe the significance and contribution of the study to current research and practice. 

**Response**

 Thank you sir for the suggestion. We have included a section on the importance of the study in the abstract. The use of abbreviations before the first use has been rectified in the manuscript.

 2.The use JCN7 was good, but the it was very old. Also, none of the cited Reference #2-4 was the direct JNC7 document. There are many updated hypertension guidelines you can refer to (1-3).

**Response**

 Thank you sir for your observation. The term prehypertension was a classification introduced in JNC VII. The main objective of the trial is to study the effect of modular intervention in halting the progress of prehypertensives to overt hypertension.

 Prehypertension was initially redefined in JNC 7. Our focus is on individuals with prehypertension as they can progress to hypertension. Since the main focus of our study is on pre-hypertensives we have used the JNC-VII report.

 3.You mentioned lifestyle modifications were important to pre-hypertension people. Please summarize the points as it was too long now.

**Response**

 Thank you, sir. We have summarized the points as per your suggestion.

 4.Please summarize the prevalence of pre-hypertension from various studies.

**Response**

 Thank you. We have incorporated the suggestion in the manuscript. 

 5.You may mention that many people with hypertension did not adhere to healthy lifestyles (4). You can use this point to link with the development of interventions for pre-hypertensive people.

**Response**

 Thank you sir We have incorporated your suggestion in the manuscript. 

 6.You described that several studies had shown the effectiveness of using multi-components to improve blood pressure and lifestyles among pre-hypertensive people, What was the value/significance of current study?

**Response**

 Thank you, sir.

 Studies using multi-component modular interventions have not been conducted previously in our study setting.

 The intervention in our study will be administered using a module that is easy to comprehend and practice with the participants.

 Modular interventions break down the process of improving health into manageable steps or modules, making it easier for individuals to implement changes gradually. The active food tracking component will self-motivate the participants to adopt healthy behaviour.

 7.Where are the villages, South, North, East or West India? Any specific reasons to select these villages?

**Response**

 The villages selected for the study are located in the southern part of India. These villages are the rural field practice area of the Department of Community Medicine, KMC Mangalore, to which the authors of the study are affiliated to.

 8.The study had been done in Dec 2022. Why to publish a study protocol, but not the study findings?

**Response**

 Analysis of the data is still underway. 

 9."For getting a block of four participants with an equal .... The participants will be allocated into either group in a batch of four." Might not be necessary.

**Response**

 Thank you, sir.  We have removed the same from the manuscript. 

 10.Please describe the development of the intervention. Any guideline to follow the development of the content? 

**Resposne**

 The intervention module is prepared based on the recommendations from WHO STEPS and National programme for prevention & control of cancer, diabetes, cardiovascular diseases & stroke (NPCDCS.

 11. What will be the expected group size? Who delivered the education? What was the interval between each session, daily, weekly?

**Response**

 The expected group size is 10 and the sessions are delivered by the principal investigator once in three months.

 12. It was unclear that there were 4 modules, but only 3 sessions. Please describe with modules will be delivered in session 1, 2, and 3.

**Response**

 The intervention tool (module) consists of four components. All the components are delivered during all three sessions.

 13. How to control subject contamination between intervention and control groups?

**Response**

 At the designing stage, we thought of assigning one of the selected villages for intervention and another selected village as control. However, this approach might result in an unequal number of prehypertensives in the intervention and control arm.

 Only one prehypertensive from every household will be recruited for the study to prevent cross-contamination.

 14. Please update blood pressure measurement. It has been suggested to measure at least twice in one measurement (1-3). 

**Response**

 Thank you sir. We have updated the measurements.

 15. Who collect the data from the participants? Who assigns the participants to groups?

**Response**

 The data is collected by the principal investigator (NF). Randomization and record keeping was done by co-investigators

 16. The use of ITT is good, but how to treat missing data?

**Response**

 The imputation technique will be used to treat the missing data. Also, the sample size was calculated considering a 10% loss to follow-up.

 The same has been incorporated in the manuscript.

 18. The change score within the group will be compared using repeated measures of ANOVA and Friedman’s test. Any test to examine the change between groups (intervention vs control)

**Response**

 Thank you, sir. Mann Whitney U tests will be used to examine the change between groups.

 The same has been included in the manuscript.

 19. Please use paragraph format. Urban-rural disparities in hypertension management have been shown globally for years (4, 5, 6). The authors need to make further discussion.

**Response**

 Thank you, sir. We have incorporated the changes as per your suggestion.

 20. There was only 71 participants assigned to intervention group, but 72 will receive the modules?

**Response**

 Thank you sir for pointing out the mismatch in the number. The same has been corrected in the manuscript.

# Reviewer report: Reviewer response for version 1

**DOI:** 10.5256/f1000research.141793.r178663

**Published:** 2023-07-04

**Authors:** Karani Magutah

**Affiliations:** 1 Moi University, Eldoret, Uasin Gishu County, Kenya

I have 2 major and 1 minor comments as here below:

1. This is a trial and will have interventions in the one group and not the other (control). Participants are drawn from the same villages/community and this is likely to have contamination where individuals in the control group may see and be influenced by those in the intervention group. There is need for careful quality control considerations to fend off likely cross-over mainly from control to intervention arm when the investigators do not want this. One way to do this is having separate but homogenous villages for intervention and the control group.
2. To avoid a likely recall bias that may be introduced by weekly tracking of diet that participants were on throughout a week that has already passed, this could either be done more regularly (like twice a week) or have participants fill daily record-logs after every meal or at the end of every day. These records can thereafter be collected at the end of the week. This will help reduce recall bias that may compromise quality of the results.
3. Physical activity (PA) should be defined such that the intensity to be considered, the length and the frequency is clear for the reader. Usually, we have mild, moderate and vigorous intensity PA. Because participation in either may yield differing outcomes, the investigators may need to in their intervention have only form form that the participants could adopt. Similarly, the number of minutes in a week that is determined by the frequency and the length for each session could be clarified.

Is the study design appropriate for the research question?

Partly

Is the rationale for, and objectives of, the study clearly described?

Yes

Are sufficient details of the methods provided to allow replication by others?

Partly

Are the datasets clearly presented in a useable and accessible format?

Yes

Reviewer Expertise:

Medical physiology and epidemiology

I confirm that I have read this submission and believe that I have an appropriate level of expertise to confirm that it is of an acceptable scientific standard, however I have significant reservations, as outlined above.

# Response

**Published:** 2023-09-06

**Authors:** Rekha Thapar

**Affiliations:** Kasturba Medical College Hospital, India

1.This is a trial and will have interventions in the one group and not the other (control). Participants are drawn from the same villages/community and this is likely to have contamination where individuals in the control group may see and be influenced by those in the intervention group. There is need for careful quality control considerations to fend off likely cross-over mainly from control to intervention arm when the investigators do not want this. **One way to do this is having separate but homogenous villages for intervention and the control group.**

**Response**

 Thank you sir for your suggestion.

 At the designing stage, we thought of assigning one of the selected villages for intervention and another selected village as control. However, this approach might result in an unequal number of prehypertensives in the intervention and control arm.

 Only one prehypertensive from every household will be recruited for the study to prevent cross-contamination.

 2. To avoid a likely recall bias that may be introduced by weekly tracking of diet that participants were on throughout a week that has already passed, this could either be done more regularly ( **like twice a week) or have participants fill daily record-logs after every meal or at the end of every day.** These records can thereafter be collected at the end of the week. This will help reduce recall bias that may compromise the quality of the results.

**Response**

 Thank you sir. We have incorporated your suggestion to record-log every meal of the participant on a daily basis.

 The manuscript has been modified accordingly

 3. Physical activity (PA) should be defined such that the intensity to be considered, the length and the frequency is clear for the reader. Usually, we have mild, moderate and vigorous intensity PA. Because participation in either may yield differing outcomes, the investigators may need to in their intervention have only form that the participants could adopt. Similarly, the number of minutes in a week that is determined by the frequency and the length for each session could be clarified.

**Response**

 The definition and recommendations for physical activity have been incorporated from the WHO-STEPS questionnaire.

 The STEPS questionnaire includes questions to assess an individual's level of physical activity. It typically covers aspects such as the frequency, duration, and intensity of physical activity, as well as the types of activities individuals engage in (e.g., walking, cycling, sports).
